# A Clinical History of the Medical and Surgical Diseases of Women

**Published:** 1878-11

**Authors:** 


### BIBLIOGRAPHICAL.

A CLINICAL HISTORY OF THE MEDICAL AND SURGICAL DISEASES ON WOMEN. By Robert Barnes, M.D., London, Censor of the Royal College lege of Physicians, etc. Second American, from the second and revised London edition, with one hundred and eighty-one illustrations, Philadelphia: Henry C. Lea. 1878.

While this standard and popular work gives the general literature of the subjects discussed, there are special practical suggestions which will be found adapted to the wants of the general practitioner as well as the specialist.

The illustrations are numerous and convey very exact representations of healthy and diseased parts as well as the appliances by which treatment is afforded.

The edition before us will doubtless prove acceptable to the profession.

